# Simultaneous incisional hernia repair and colorectal surgery: one or two-step procedure?

**DOI:** 10.1007/s10029-024-03164-z

**Published:** 2024-09-26

**Authors:** M. Verdaguer-Tremolosa, V. Rodrigues-Gonçalves, M. P. Martínez-López, J. L. Sánchez-García, M. López-Cano, in representation of EVEREG group

**Affiliations:** 1 Department of Surgery, UD of Medicine of Vall d’Hebron, Universitat Autònoma de Barcelona, General and Digestive Surgery Department, Abdominal Wall Surgery Unit, Hospital Universitari Vall d’Hebrón, Passeig Vall d’Hebron 119, 08035 Barcelona, Spain; 2 General and Digestive Surgery Department, Colorectal Surgery Unit, Hospital Universitari Vall d’Hebrón, Universitat Autònoma de Barcelona, Passeig Vall d’Hebron 119-129, 08035 Barcelona, Spain

**Keywords:** Incisional, Hernia, Colorectal, Concomitant, Surgery

## Abstract

**Purpose:**

Patients requiring colorectal surgery in the context of an incisional hernia are common, but it is not clear whether the repair should be performed as a single or two-step surgery. Our aim was to evaluate complications after concomitant abdominal wall repair and colorectal surgery compared to those after incisional hernia repair alone.

**Methods:**

Adult patients who underwent elective incisional hernia surgery from 2012–2022 from the EVEREG registry were included. Patients who underwent midline incisional hernia repair as a single procedure and patients who underwent midline incisional hernia repair concomitant with colorectal surgery were included. The primary outcome was surgical site infection (SSI). The secondary outcomes were the Clavien–Dindo classification grade, in-hospital mortality and recurrence.

**Results:**

A total of 7783 patients were included: 256(3.3%) who underwent concomitant surgery and 7527(96.7%) who underwent only midline incisional hernia repair. The first group included more comorbid patients and complex hernias. SSI was found in 55.4% of patients who underwent simultaneous surgery compared to 30.7% of patients who underwent hernia repair alone (*P* = 0.000). Multivariate analysis revealed that the risk factors for SSI were BMI (OR = 1.07, 95% CI 1.02–1.11; *P* = 0.004), smoking (OR = 1.89, 95% CI 1.12–3.19; *P* = 0.017), transverse diameter (OR = 1.06, 95% CI 1.01–1.11; *P* = 0.017), component separation (OR = 1.996, 95% CI 1.25–3.08; *P* = 0.037) and clean-contaminated and contaminated surgeries(OR = 3.86, 95% CI 1.36–10.66; *P* = 0.009). Higher grades of Clavien–Dindo (*P* = 0.001) and mortality rates (*P* < 0.001) were found in the colorectal surgery group, although specific risk factors were detected. No differences were observed in terms of recurrence (*P* = 0.104).

**Conclusions:**

Concomitant surgery is related to greater risk of complications, especially in patients with comorbidities and complex hernias. In properly selected cases, simultaneous procedures can yield satisfactory results.

## Introduction

Incisional hernia has been described in 12.8% of patients after abdominal surgery, although some risk factors can increase that percentage and aggravate the described recurrence rates of 23–50% after hernia repair [1]. Some patients who require colorectal surgery present with a concomitant incisional hernia, but it is not clear whether the repair should be performed via a single approach or a two-step procedure, although mesh can be safely used in clean-contaminated environments [2–4].

Some authors have suggested that performing these concomitant procedures is safe [5–7], but others have described a greater risk of complications [8–11]. The advantages of a single operation include cost-effectiveness, the avoidance of comorbidities from two separate surgeries and patient satisfaction. However, there are concerns regarding mesh infection and recurrence risk, especially in complex abdominal wall repairs or demanding colorectal surgery.

Since the literature is scarce and controlled trials are difficult to perform due to the heterogeneity of hernias and colorectal procedures, available data from registries are vital [9, 10]. These data represent “real-world evidence”, the daily clinical practice in the general population and can be instruments of quality control and constructive feedback for surgeons or health-care systems, as well as revealing surgical tendencies and providing long-term information about issues rarely observed in controlled trials, especially for infrequent variables or outcomes [12–14].

Our aim was to evaluate the rates of complications and recurrence after concomitant abdominal wall repair and colorectal surgery compared to those after incisional hernia repair only according to data from the EVEREG registry.

## Materials and methods

The EVEREG database (http://www.evereg.es/) includes nearly 200 Spanish hospitals. The incisional hernia registry is anonymous and is maintained by surgeons who are interested in abdominal wall surgery. This online national prospective audited register collects data on patient and hernia characteristics, and information about surgery, postoperative complications and follow-up [15, 16].

Adult patients (≥ 18 years old) who underwent elective incisional hernia surgery from the EVEREG registry between July 2012 and January 2022 were included. Patients who underwent midline incisional hernia repair as a single procedure and patients who underwent midline incisional hernia repair concomitant with surgery involving the colon or rectum were included. Colorectal procedures include colectomies for benign or malignant disease and stoma reversal.

Patients who underwent different gastrointestinal procedures, different types of hernias rather than midline hernias, emergency surgeries or other clean-contaminated, contaminated or dirty hernia repairs were excluded.

Patient demographic data (sex, age, body mass index (BMI)) and comorbidities (diabetes status, chronic obstructive pulmonary disease (COPD), smoking status and American Society of Anesthesiologists (ASA)) were collected.

Hernia characteristics including the transverse diameter of the defect and the European Hernia Society classification [17] were registered, as well as wound contamination according to the US Centers for Disease Control and Prevention (CDC) [18, 19] or recurrent hernia. Hernia type according to the European Hernia Society (EHS) classification was grouped as supraumbilical (types M1 and M2), umbilical (M3), infraumbilical (M4 and M5) or combined if more than one was present. Hernias meeting the criteria established by Slater et al. [20] were considered complex.

Data from surgeries such as surgical approach, surgical technique, component separation (CS), and the number and position of mesh used, were recorded. Information about additional procedures performed and the presence of a surgeon with experience in abdominal wall surgery was collected. Information about postoperative complications, recurrence, mortality, hospital stay and length of follow-up was collected. Clavien–Dindo classification [21] degrees were grouped as mild (I-II) or severe (III-V).

This cohort study was conducted in accordance with the STROBE and RECORD statement [22, 23]. The primary outcome was surgical site infection (SSI). The secondary outcomes were the Clavien–Dindo grade, mortality during hospital admission and recurrence at the maximum follow-up.

A subgroup analysis was conducted on patients who underwent surgery at Vall d’Hebron University Hospital by a certified colorectal surgeon and a certified abdominal wall surgeon.

### Statistical analysis

Continuous variables are expressed as the mean, standard deviation (SD), minimum and maximum. Categorical variables are represented as frequencies and percentages. Continuous variables were compared using Student’s *t* test or the Mann–Whitney *U* test, and categorical variables were compared by the *X*^2^ test or Fisher’s exact test, according to the conditions of application. The normality of the distribution of quantitative variables was evaluated using the Kolmogorov–Smirnov test.

A multivariate binary logistic regression model was used to identify risk factors for each outcome. Each model initially included variables with a P-value less than 0.1 obtained in the bivariant analysis. Nonsignificant variables in this model were then excluded, taking into account the confounding factors and potential interactions according to investigators and a statistical expert. The results show odds-ratios (OR), and confidence intervals (95%) as well as P-values from Wald’s test. For all the statistical analyses, P < 0.05 was considered significant. For multivariate analysis, variables with scarce data increasing model instability were excluded according to statistical expert recommendations. The statistical software used for the analysis was SPSS 26.0 (IBM Corp.).

## Results

A total of 7783 patients were included; 256(3.3%) who underwent concomitant surgery of the abdominal wall and the colon or rectum, and 7527(96.7%) who underwent midline incisional hernia repair via a single procedure.

The demographic and hernia characteristics of the population are described in Table 1. A greater proportion of men, older patients, COPD patients and patients with higher ASA scores were found in the concomitant colorectal surgery group. A higher proportion of multiple midline defects and a wider transverse diameter of the hernia defect were found in this group.

**Table 1:** Patient and hernia characteristics

Variables	Total	Hernia repair with colorectal surgery	Hernia repair only	*P*
**Age** (years) [mean, SD]	69.66 (13.06)	71.73 (12.43)	69.6 (13.08)	**0.009**
**Sex** (n, %) Male Female	3505 (45.1) 4262 (54.9)	137 (53.5) 119 (46.5)	3368 (44.8) 4143 (55.2)	**0.006**
**BMI** (kg/m^2^) [mean, SD]	30.12 (5.35)	29.71 (5.27)	30.13 (5.35)	0.212
**Diabetes** (n, %)	1595 (20.6)	62 (24.2)	1533 (20.4)	0.142
**COPD** (n, %)	1121 (14.5)	52 (20.3%)	1069 (14.3)	**0.007**
**Smoking** (n, %)	1728 (22.2)	49 (19.1)	1679 (22.4)	0.224
**ASA score** (n, %) I–II III/IV	5631 (74.1) 1967 (25.9)	173 (67.8) 82 (32.2)	5458 (74.3) 1885 (25.7)	**0.020**
**Hernia type** (n, %) M1-M2 M3 M4-M5 Combined	1607 (20.6) 1940 (24.9) 963 (12.4) 3273 (42.1)	16 (6.3) 14 (5.5) 16 (6.3) 210 (82)	1591 (21.1) 1926 (25.6) 947 (12.6) 3063 (40.7)	** < 0.001**
**Recurrent hernia** (n, %)	1807 (23.3)	48 (18.8)	1759 (23.5)	0.080
**Transverse diameter** (cm) [mean, SD]	7.1 (4.46)	11.41 (5.69)	7 (4.38)	** < 0.001**

*BMI* body mass index, *COPD* chronic obstructive pulmonary disease, *ASA* American Society of Anesthesiologists

The surgical and follow-up characteristics are shown in Table 2. Minimally invasive surgery (MIS) was more frequent in patients who underwent a single procedure. The presence of a surgeon with experience in abdominal wall repairs and CS techniques were more common in the concomitant procedures group. Anterior CS was predominant in the colorectal group (64.4%). Hernia repair and stoma reversal were performed in 105 patients (41%).

**Table 2:** Surgery characteristics and postoperative follow-up

Variables	Total	Hernia repair with colorectal surgery	Hernia repair only	*p*
**Approach** (n, %) Open Laparoscopy	6876 (90.2) 746 (9.8)	252 (98.8) 3 (1.2)	6624 (89.9) 743 (10.1)	** < 0.001**
**Abdominal wall experienced surgeon** (n, %)	2390 (54.7)	127 (71.3)	2263 (54)	** < 0.001**
**Component separation** (n, %)	1151 (15.1)	130 (52)	1021 (13.9)	** < 0.001**
**CDC classification** (n, %) I II III IV	5777 (93.8) 322 (5.2) 56 (0.9) 6 (0.1)	19 (11.7) 117 (71.8) 27 (16.6) 0	5758 (96) 205 (3.4) 29 (0.5) 6 (0.1)	** < 0.001**
**Use a mesh** (n, %)	7526 (98.6)	246 (96.1)	7280 (98.7)	**0.001**
**Number of meshes** [mean, SD]	1.11 (0.31)	1.17 (0.41)	1 (0)	** < 0.001**
**Mesh type** (n, %) Synthetic Biosynthetic Biological	7437 (99.3) 22 (0.3) 28 (0.4)	230 (94.3) 12 (4.9) 2 (0.8)	7207 (99.5) 10 (0.1) 26 (0.4)	** < 0.001**
**Mesh position** (n, %) Onlay Inlay Retromuscular Preperitoneal Intraperitoneal	2954 (39.6) 266 (3.6) 1778 (23.8) 1015 (13.6) 1442 (19.3)	122 (52.1) 7 (3) 75 (32.1) 12 (5.1) 18 (7.7)	2832 (39.2) 259 (3.6) 1703 (23.6) 1003 (13.9) 1424 (19.7)	** < 0.001**
**SSI** (n, %)	181 (34.2)	41 (55.4)	140 (30.7)	** < 0.001**
**Type of SSI** (n, %) Superficial Deep Organ/space	27 (51.9) 7 (13.5) 18 (34.6)	9 (47.4) 3 (15.8) 7 (36.8)	18 (54.5) 4 (12.1) 11 (33.3)	0.868
**Clavien-Dindo** (n, %) I–II III–V	429 (78.3) 119 (21.7)	39 (61.9) 24 (38.1)	390 (80.4) 95 (19.6)	**0.001**
**Postoperative mortality** (n, %)	20 (0.5)	6 (3.4)	14 (0.4)	** < 0.001**
**Length of hospital stay** (days) [mean, SD]	4.34 (9.03)	11.3 (10.97)	4.12 (8.88)	** < 0.001**
**Recurrence** (n, %)	337 (31.4)	18 (42.9)	319 (31)	0.104
**Follow-up** (months) [mean, SD]	10.1 (9.21)	10.13 (7.73)	10.10 (9.26)	0.239

*BMI* body mass index, *COPD* chronic obstructive pulmonary disease, *ASA* American Society of Anesthesiologists, *SSI* surgical site infection

According to the CDC classification, clean surgery was most common in the hernia repair group, and the use of combined meshes was more common in the colorectal group, which included more biosynthetic meshes (Table 2). Supraponeurotic and retromuscular repairs were predominant in the colorectal group while preperitoneal and intraperitoneal prostheses were mostly found in the other group. Additional procedures (cholecystectomy, dermolipectomy, parastomal hernia repair, mesh excision, extended adhesiolysis, ostomy creation or gynecological interventions) were performed in 31.3% of patients from the concomitant colorectal surgery group.

SSI was more frequent in the concomitant colorectal surgery group (P < 0.001). No differences were found in terms of superficial, deep or organ-space infection between groups. Regarding the Clavien–Dindo classification, higher grades were found in the group that underwent colorectal surgery. Postoperative mortality was greater in this group, although scarce data are available (Table 2).

### SSI

SSI was associated with BMI and smoking (Table 3). Although hernia type was not related to SSI, a greater transverse diameter of the defect and the need for CS were associated to SSI. Forty percent of patients with anterior CS presented with SSI while 13% presented with posterior CS (P = 0.004). Higher CDC classification grades and an association with colorectal procedures were observed in the SSI group. In the last cohort, 55.4% of patients (41/181) presented with SSI, whereas 30.7% of patients who underwent hernia repair alone (140/456) presented with SSI. Stoma reversal was not associated with worse outcomes. Biosynthetic and biological meshes were associated with SSI (P = 0.004), although they could be preferred in contaminated fields. The mesh position did not influence the SSI. The SSI was related to higher degrees of Clavien–Dindo classification, but no differences were observed in terms of in-hospital mortality or recurrence.

**Table 3:** Analysis of surgical site infection

Variable	No SSI	SSI	*p*	Multivariable OR (CI 95%)	*p*
**Age** (years) [mean, SD]	73.69 (12.86)	71.93 (11.25)	0.057		
**Sex** (n, %) Male Female	197 (56.4) 152 (43.6)	94 (51.9) 87 (48.1)	0.322		
**BMI** (kg/m^2^) [mean, SD]	29.03 (5.12)	30.67 (5.83)	**0.004**	1.07 (1.02–1.11)	**0.004**
**Diabetes** (n, %)	83 (23.9)	49 (27.1)	0.417		
**COPD** (n, %)	79 (22.6)	46 (25.4)	0.475		
**Smoking** (n, %)	69 (19.8)	54 (29.8)	**0.009**	1.89 (1.12–3.19)	**0.017**
**ASA score** (n, %) I-II III/IV	215 (62.1) 131 (37.9)	104 (57.5) 77 (42.5)	0.297		
**Hernia type** (n, %) M1-M2 M3 M4-M5 Combined	44 (12.6) 31 (8.9) 43 (12.3) 231 (66.2)	13 (7.2) 17 (9.4) 20 (11) 131 (72.4)	0.248		
**Recurrent hernia** (n, %)	101 (28.9)	52 (28.7)	0.960		
**Transverse diameter** (cm) [mean, SD]	9.05 (4.7)	11.46 (5.61)	** < 0.001**	1.06 (1.01–1.11)	**0.017**
**Group procedure** (n, %) CCR and hernia Hernia	33 (9.5) 316 (90.5)	41 (22.7) 140 (77.3)	** < 0.001**	0.829 (0.49–3.27)	0.630
**Approach** (n, %) Open Laparoscopy	336 (96.8) 11 (3.2)	178 (98.3) 3 (1.7)	0.399		
**Abdominal wall experienced surgeon** (n, %)	123 (58.3)	62 (68.9)	0.084		
**Component separation** (n, %)	95 (27.5)	78 (43.8)	** < 0.001**	1.966 (1.25–3.08)	**0.037**
**Stoma reversal** (n, %)	17 (51.5)	15 (36.6)	0.198		
**CDC classification** (n, %) I II III IV	234 (83) 39 (13.8) 8 (2.8) 1 (0.4)	87 (62.1) 40 (28.6) 13 (9.3) 0	** < 0.001**	3.86 (1.36–10.66)	**0.009**
**Use a mesh** (n, %)	346 (99.1)	175 (96.7)	0.069		
**Number of meshes** [mean, SD]	1.19 (0.4)	1.27 (0.46)	0.052		
**Mesh type** (n, %) Synthetic Biosynthetic Biological	339 (99.1) 2 (0.6) 1 (0.3)	164 (94.3) 5 (2.9) 5 (2.9)	**0.004**	0.163 (0.016–1.68)	0.128
**Mesh position** (n, %) Onlay Inlay Retromuscular Preperitoneal Intraperitoneal	127 (37.4) 7 (2.1) 123 (36.2) 25 (7.4) 58 (17.1)	77 (44.8) 4 (2.3) 57 (33.1) 11 (6.4) 23 (13.4)	0.555		
**Clavien-Dindo** (n, %) I-II III-V	224 (78.3) 62 (21.7)	46 (54.8) 38 (45.2)	** < 0.001**		
**Postoperative mortality** (n, %)	8 (2.3)	7 (3.9)	0.292		
**Length of hospital stay** (days) [mean, SD]	9.39 (10.1)	18.5 (22.35)	** < 0.001**		
**Recurrence** (n, %)	22 (26.5)	18 (28.6)	0.782		
**Follow-up** (months) [mean, SD]	9.11 (8.64)	12.12 (8.9)	**0.001**		

*BMI* body mass index, *COPD* chronic obstructive pulmonary disease, *ASA* American Society of Anesthesiologists, *CCR* concomitant colorectal procedure, *SSI* surgical site infection

Multivariate analysis revealed that the risk factors for SSI were BMI (OR = 1.07, 95% CI 1.02–1.11; P = 0.004), smoking (OR = 1.89, 95% CI 1.12–3.19; P = 0.017), transverse diameter (OR = 1.06, 95% CI 1.01–1.11; P = 0.017), CS (OR = 1.996, 95% CI 1.25–3.08; P = 0.037) and clean-contaminated and contaminated fields (OR = 3.86, 95% CI 1.36–10.66; P = 0.009).

### Clavien–Dindo classification

Higher COPD incidence and ASA scores were associated with higher Clavien–Dindo grades (Table 4). Moreover, concomitant surgery was associated with higher-grade complications (P = 0.001). Higher grades were observed for clean-contaminated (32%) and contaminated fields (52.6%). Similarly, 31% of CS were associated with high-grade complications compared to 17.5% of cases who did not require this technique.

**Table 4:** Analysis of Clavin-Dindo classification

Variable	Clavien I-II	Clavien III-V	*p*	Multivariable OR (CI 95%)	*p*
**Age** (years) [mean, SD]	71.86 (12.71)	73.73 (12.2)	0.163		
**Sex** (n, %) Male Female	227 (52.9) 202 (47.1)	65 (54.6) 54 (45.4)	0.741		
**BMI** (kg/m^2^) [mean, SD]	29.98 (5.48)	29.64 (5.7)	0.382		
**Diabetes** (n, %)	105 (24.5)	34 (28.8)	0.338		
**COPD** (n, %)	82 (19.1)	41 (34.5)	** < 0.001**	2.195 (1.16–4.15)	**0.015**
**Smoking** (n, %)	86 (20)	23 (19.3)	0.862		
**ASA score** (n, %) I–II III/IV	290 (68.2) 135 (31.8)	62 (52.1) 57 (47.9)	**0.001**	1.093 (0.59–2)	0.773
**Hernia type** (n, %) M1-M2 M3 M4-M5 Combined	64 (14.9) 42 (9.8) 56 (13.1) 267 (62.2)	14 (11.8) 10 (8.4) 8 (6.7) 87 (73.1)	0.125		
**Recurrent hernia** (n, %)	119 (27.7)	44 (37)	0.051	1.91 (1.04–3.52)	**0.036**
**Transverse diameter** (cm) [mean, SD]	8.76 (4.79)	10.86 (5.95)	**0.005**	1.021 (0.96–1.08)	0.494
**Group procedure** (n, %) CCR and hernia Hernia	39 (9.1) 390 (90.9)	24 (20.2) 95 (79.8)	**0.001**	1.719 (0.57–5.1)	0.333
**Approach** (n, %) Open Laparoscopy	385 (90.2) 42 (9.8)	111 (93.3) 8 (6.7)	0.298		
**Abdominal wall experienced surgeon** (n, %)	153 (59.5)	63 (70)	0.078		
**Component separation** (n, %)	110 (25.8)	50 (42.7)	** < 0.001**	1.232 (0.66–2.29)	0.511
**Stoma reversal** (n, %)	16 (41)	7 (29.2)	0.342		
**CDC classification** (n, %) I II III IV	276 (83.1) 47 (14.2) 9 (2.7) -	59 (64.1) 23 (25) 10 (10.9) -	** < 0.001**	4.7 (1.21–18.2)	**0.025**
**Use a mesh** (n, %)	421 (98.1)	117 (98.3)	1		
**Number of meshes** [mean, SD]	1.19 (0.4)	1.3 (0.46)	**0.017**	1.098 (0.56–2.16)	0.785
**Mesh type** (n, %) Synthetic Biosynthetic Biological	411 (98.6) 4 (1) 2 (0.5)	112 (95.7) 3 (2.6) 2 (1.7)	0.101		
**Mesh position** (n, %) Onlay Inlay Retromuscular Preperitoneal Intraperitoneal	156 (37.4) 12 (2.9) 140 (33.6) 356 (8.4) 74 (17.7)	35 (30.4) 3 (2.6) 44 (38.3) 6 (5.2) 27 (23.5)	0.343		
**SSI** (n, %)	46 (17)	38 (38)	** < 0.001**	2.371 (1.24–4.56)	**0.01**
**Type of SSI** (n, %) Superficial Deep Organ/space	21 (84) 2 (8) 2 (8)	6 (22.2) 4 (18.5) 16 (59.3)	** < 0.001**		
**Length of hospital stay** (days) [mean, SD]	8.24 (10.01)	19.8 (23.6)	** < 0.001**		
**Recurrence** (n, %)	36 (34.6)	8 (36.4)	0.876		
**Follow-up** (months) [mean, SD]	10.41 (9.36)	8.82 (8.13)	0.242		

*BMI* body mass index, *COPD* chronic obstructive pulmonary disease, *ASA* American Society of Anesthesiologists, *CCR* concomitant colorectal procedure, *SSI* surgical site infection

According to multivariate analysis, COPD (OR = 2.195, 95% CI 1.16–4.15; P = 0.015), recurrent hernia (OR = 1.91, 95% CI 1.04–3.52; P = 0.036), contaminated field (OR = 4.7, 95% CI 1.21–18.2; P = 0.025) and SSI (OR = 2.371, 95% CI 1.24–4.56; P = 0.010) were associated with higher grades of Clavien–Dindo classification.

### Mortality

Mortality was significantly greater in older and comorbid patients (Table 5). The postoperative mortality rate of patients who underwent concurrent colorectal surgery was 3.4%, whereas that of patients who underwent hernia repair alone was 0.4%. Stoma reversal was not associated with increased mortality. Complex hernias were related to increased mortality. Approximately 1.5% of patients with CS died during the postoperative period, compared to 0.3% of those who did not require this technique (P < 0.001). The clean-contaminated and contaminated field groups presented higher mortality rates (P < 0.001). Mesh was related to lower mortality, but it was used in the majority of patients. Although colorectal surgery was previously related to SSI, the presence or type of SSI did not correlate with mortality.

**Table 5:** Analysis of postoperative mortality

Variable	Postoperative mortality		*p*	Multivariable OR (CI 95%)	*p*
	**No**	**Yes**			
**Age** (years) [mean, SD]	71.17 (12.9)	83.3 (8.28)	** < 0.001**	1.237 (1.056–1.45)	**0.008**
**Sex** (n, %) Male Female	1720 (45.4) 2071 (54.6)	12 (60) 8 (40)	0.190		
**BMI** (kg/m^2^) [mean, SD]	30.11 (5.53)	30.82 (4.92)	0.393		
**Diabetes** (n, %)	789 (20.8)	8 (40)	**0.035**	0.382 (0.041–3.55)	0.398
**COPD** (n, %)	514 (14.3)	13 (65)	** < 0.001**	14.8 (1.33–165)	**0.028**
**Smoking** (n, %)	814 (21.5)	2 (10)	0.281		
**ASA score** (n, %) I-II III/IV	2714 (71.7) 1071 (28.3)	5 (25) 15 (75)	** < 0.001**	0.864 (0.91–8.2)	0.899
**Hernia type** (n, %) M1-M2 M3 M4-M5 Combined	825 (21.8) 653 (17.2) 559 (14.7) 1755 (46.3)	3 (15) 0 0 17 (85)	**0.003**	1.45 (0.156–13.54)	0.740
**Recurrent hernia** (n, %)	922 (24.3)	12 (60)	** < 0.001**	3.75 (0.55–25.4)	0.176
**Transverse diameter** (cm) [mean, SD]	8.25 (4.71)	12.81 (5.98)	**0.001**	1.089 (0.93–1.27)	0.282
**Group procedure** (n, %) CCR and hernia Hernia	173 (4.6) 3619 (95.4)	6 (30) 14 (70)	** < 0.001**		
**Approach** (n, %) Open Laparoscopy	3385 (89.3) 404 (10.7)	19 (95) 1 (5)	0.512		
**Abdominal wall experienced surgeon** (n, %)	568 (53.7)	7 (87.5)	0.076		
**Component separation** (n, %)	641 (16.9)	10 (50)	** < 0.001**	1.99 (0.34–11.4)	0.440
**Stoma reversal** (n, %)	68 (39.3)	0	0.084		
**CDC classification** (n, %) I II III IV	2335 (92.4) 159 (6.3) 28 (1.1) 4 (0.2)	3 (25) 7 (58.3) 2 (16.7) 0	** < 0.001**	31.6 (4–250)	**0.001**
**Use a mesh** (n, %)	3736 (98.5)	18 (90)	**0.035**		
**Number of meshes** [mean, SD]	1.13 (0.33)	1.47 (0.51)	** < 0.001**	2.01 (0.31–13.7)	0.450
**Mesh type** (n, %) Synthetic Biosynthetic Biological	3868 (99.1) 14 (0.4) 21 (0.6)	18 (100) 0 0	1		
**Mesh position** (n, %) Onlay Inlay Retromuscular Preperitoneal Intraperitoneal	1596 (43) 100 (2.7) 802 (21.6) 392 (10.6) 820 (22.1)	6 (33.3) 2 (11.1) 5 (27.8) 0 5 (27.8)	0.119		
**SSI** (n, %)	171 (33.6)	7 (46.7)	0.292		
**Clavien-Dindo** (n, %) I-II III-V	427 (81.3) 98 (18.7)	0 20 (20)	** < 0.001**		
**Length of hospital stay** (days) [mean, SD]	5.49 (11.69)	16.65 (25.18)	**0.001**		

*BMI* body mass index, *COPD* chronic obstructive pulmonary disease, *ASA* American Society of Anesthesiologists, *CCR* concomitant colorectal procedure, *SSI* surgical site infection

Multivariate analysis revealed higher age (OR = 1.237, 95% CI 1.056–1.45; P = 0.008), COPD (OR = 14.8, 95% CI 1.33–165; P = 0.028) and higher grade of CDC classification (OR = 31.6, 95% CI 4–250; P = 0.001) as risk factors for mortality.

### Recurrence

The incidence of recurrence was greater in older patients, those with an increased BMI and those with a lower ASA grade (Table 6). Complex hernias and MIS were associated with a greater percentage of recurrence. Simultaneous colorectal surgery and concomitant stoma reversal did not influence this outcome. A slightly greater number of meshes was detected in the nonrecurrence group. The recurrence rate was 55.8% for the inlay position group, followed by the preperitoneal group (40.2%). Lower recurrence rates were found for the retromuscular (25.3%) and intraperitoneal positions (24.3%) (P < 0.001).

**Table 6:** Analysis of recurrence at maximum follow-up

Variable	No recurrence	Recurrence	*p*	Multivariable OR (CI 95%)	*p*
**Age** (years) [mean, SD]	72.17 (12.29)	70.09 (11.64)	**0.004**	0.992 (0.97–1)	0.252
**Sex** (n, %) Male Female	351 (46.1) 411 (53.9)	154 (45.7) 183 (54.3)	0.911		
**BMI** (kg/m^2^) [mean, SD]	30.24 (5.72)	30.92 (5.22)	**0.012**	1.027 (0.99–1.05)	0.089
**Diabetes** (n, %)	174 (22.8)	67 (19.9)	0.286		
**COPD** (n, %)	104 (13.6)	56 (16.7)	0.191		
**Smoking** (n, %)	154 (20.2)	78 (23.1)	0.272		
**ASA score** (n, %) I–II III/IV	504 (66.3) 256 (33.7)	260 (77.6) 75 (22.4)	** < 0.001**	0.743 (.051–1.08)	0.118
**Hernia type** (n, %) M1–M2 M3 M4–M5 Combined	147 (19.3) 130 (17) 108 (14.2) 378 (49.5)	71 (21.1) 88 (26.1) 42 (12.5) 136 (40.4)	**0.002**	1.044 (0.67–1.63)	0.850
**Recurrent hernia** (n, %)	196 (25.7)	117 (34.7)	**0.002**	1.522 (1.08–2.13)	**0.015**
**Transverse diameter** (cm) [mean, SD]	8.56 (4.75)	7 (4.56)	** < 0.001**	0.947 (0.91–0.99)	**0.010**
**Group procedure** (n, %) CCR and hernia Hernia	52 (6.8) 711 (93.2)	18 (5.3) 319 (94.7)	0.356		
**Approach** (n, %) Open Laparoscopy	701 (91.9) 62 (8.1)	294 (87.2) 43 (12.8)	**0.016**	2.228 (1.18–4.22)	**0.014**
**Abdominal wall experienced surgeon** (n, %)	87 (40.5)	92 (43.6)	0.512		
**Component separation** (n, %)	153 (20.2)	33 (9.8)	** < 0.001**	0.621 (0.37–1.04)	0.068
**Stoma reversal** (n, %)	29 (55.8)	9 (50)	0.627		
**CDC classification** (n, %) I II III IV	526 (90.5) 48 (8.3) 6 (1) 1 (0.2)	249 (93.6) 16 (6) 1 (0.4) 0	0.521		
**Use a mesh** (n, %)	752 (98.6)	327 (97)	0.088		
**Number of meshes** [mean, SD]	1.15 (0.362)	1.1 (0.303)	**0.04**	0.837 (0.48–1.44)	0.521
**Mesh type** (n, %) Synthetic Biosynthetic Biological	738 (98.5) 7 (0.9) 4 (0.5)	320 (98.8) 3 (0.9) 1 (0.3)	1		
**Mesh position** (n, %) Onlay Inlay Retromuscular Preperitoneal Intraperitoneal	291 (39.3) 19 (2.6) 165 (22.3) 67 (9) 199 (26.9)	135 (41.7) 24 (7.4) 56 (17.3) 45 (13.9) 64 (19.8)	** < 0.001**	2.873 (1.22–6.78)	**0.016**
**SSI** (n, %)	45 (42.5)	18 (45)	0.782		
**Clavien-Dindo** (n, %) I–II III–V	68 (82.9) 14 (17.1)	36 (81.8) 8 (18.2)	0.876		
**Length of hospital stay** (days) [mean, SD]	5.76 (11.2)	4.82 (8.93)	** < 0.001**		
**Follow-up** (months) [mean, SD]	15.72 (9.56)	13.35 (8.59)	**0.001**		

*BMI* body mass index, *COPD* chronic obstructive pulmonary disease, *ASA* American Society of Anesthesiologists, *CCR* concomitant colorectal procedure, *SSI* surgical site infection

According to multivariate analysis, recurrent hernia (OR = 1.522, 95% CI 1.08–2.13), wider transverse diameter of the hernia (OR = 0.947, 95% CI 0.91–0.99), MIS (OR = 2.228, 95% CI 1.18–4.22) and mesh position (OR = 2.873, 95% CI 1.22–6.78) were related to greater risk of recurrence (P < 0.02 for each comparison).

### Subgroup analysis

Patients who underwent concomitant procedures performed by a colorectal expert surgeon and an abdominal wall expert surgeon (n = 33) presented greater rates of SSI than did those who underwent hernia repair alone (58.8% vs 30.7%, P = 0.014), but no differences were found in terms of the Clavien–Dindo classification (P = 0.112) or recurrence (15.2% vs. 31%, P = 0.055). Postoperative mortality was not observed in this subgroup, and no mesh was explanted.

In the case of ostomy closure in this subgroup (n = 16), more combined defects (P < 0.001) and CS (P = 0.002) were detected. Compared to the group with exclusive hernia repair, no differences were detected in terms of SSI (P = 0.707), Clavien–Dindo classification (P = 0.339) or postoperative mortality (P = 1). However, higher recurrence rates were found in the group with exclusive hernia repair (6.3% vs. 31%, P = 0.030).

## Discussion

Concomitant abdominal wall and colorectal surgery is a controversial therapeutic strategy with conflicting evidence [5, 6, 8–10, 24]. This procedure allows comprehensive treatment of two pathologies in a single procedure; it can be cost-effective and improve patient satisfaction by reducing the risks of two separate surgeries [5–7]. Nevertheless, concerns regarding this procedure include surgical complexity, infection risk, postoperative complications (including anastomotic leakage), mortality and hernia recurrence [8–11]. However, when opting for two separate procedures, various reasons might lead to the second surgery never taking place, and the patient ends up living with an incapacitating hernia or requiring an emergent operation [5]. The decision to choose a concomitant procedure rather than just a “single-step” intervention could depend on the individual complexity of the patient (i.e., comorbidities), the hernia (i.e., technical complexity) or the visceral surgery itself (i.e., colorectal).

Current evidence is based on observational studies (retrospective) with heterogeneous groups and a limited number of patients, some of whom reported a foreseeable higher complication rate for comorbid patients [8] and no data about potentially relevant outcomes, such as mesh explants. Colorectal and abdominal wall procedures are heterogeneous, thus, some studies include different visceral resections (i.e. rectum or colon) [10], stoma reversal [8], parastomal hernias, or complex abdominal wall repairs [24], diversifying the surgical techniques and diluting the applicability of the results. The use of MIS (classical laparoscopic approach, robotic platforms or hybrid procedures), which can reduce the incidence of SSI, remains to be explored.

Our study revealed worse outcomes for concomitant procedures. However, this group included more patients with comorbidities and complex hernias, which are well-recognized risk factors for complications after hernia and colorectal surgery [25–28]. Additional procedures and wider defects were observed in the colorectal surgery group, which could be associated with more symptomatic hernias forcing its repair, or it might interfere with the colorectal surgery; this could also explain the presence of an experienced abdominal wall surgeon performing demanding techniques, mainly in complex cases. Despite concomitant procedures, no differences were observed in terms of recurrence, and simultaneous stoma reversal presented comparable outcomes.

Moreover, simultaneous procedures were associated with increased rates of SSI, which was correlated with the CDC classification of surgery and patient risk factors. Even in clean-contaminated or selected contaminated environments, the use of a mesh has been proven to be safe and effective [2, 3, 29], and selected mesh types could better tolerate an infection without the need for explants [30]. Nonetheless, exclusive hernia repair reported a high proportion of SSI (30%), which is unusual for clean surgeries [31], although missing data could have influenced that percentage.

Similar findings were detected for the Clavien–Dindo classification, but the colorectal group had more COPD, recurrent hernias and higher CDC classification grades. This classification does not exclusively represent complications related to surgery; it also evaluates the treatment of systemic complications potentially influenced by comorbid patients. Prehabilitation programs led by multidisciplinary teams could optimize patients’ comorbidities and improve these outcomes [5, 24]. Protocols for prompt detection of anastomotic leakage can allow for the treatment of complications at an early stage and promote conservative strategies [32, 33].

Recognized hernia risk factors and surgical techniques influence recurrence rates [25]. Some other factors might be missing in our analysis due to scarce data. The presence of an experienced abdominal wall surgeon seemed to have no effect on recurrence, although we cannot exclude the possibility that it was preferred for complex defects or comorbid patients. According to the subgroup analysis from our center, patients who underwent surgery by experts presented improved outcomes, even for stoma reversal, although limited data were available.

Limitations of our study include those of registries, mainly in terms of loss to follow-up [14, 34]. It is unknown whether patients received prehabilitation strategies or whether a shared decision-making model was applied. It was not possible to claim that colorectal procedures were performed by experts in colorectal surgery, and the definition of “surgeon with experience” in abdominal wall surgery does not imply its certification. We could not compare our cohort with similar patients receiving only colorectal surgery to evaluate complication rates independent of hernia repair. The complexity of colorectal surgery was not evaluated (i.e., high-risk anastomosis, radiation or enterocutaneous fistula), and it could influence the risk of complications, highlighting the importance of case selection. Patients who received two different procedures were not evaluated, and it is not possible to know their progression or if they received a second-step surgery. The costs of the procedures and emergency cases were not evaluated.

Further studies should assess prehabilitation programs, both in one- and two-step procedures. Hernia repair and some colorectal procedures, in the context of benign disease, can be deferred; they allow for comorbidity optimization and preparation of the abdominal wall if needed (i.e., Botulinum toxin) with the aim of improving outcomes, especially if performed in reference centers with expert surgeons.

## Conclusions

Concomitant surgery is related to greater risk of SSI and complications, especially in comorbid patients and complex hernias. However, in properly selected cases and in expert hands, simultaneous procedures present satisfactory results, especially in stoma reversal cases. Prehabilitation programs to improve patients’ conditions and share decision-making models should be applied.
